# Methodological issues in consciousness research: theory comparison, the role of empirical evidence, and a replication crisis

**DOI:** 10.3389/fpsyg.2025.1633907

**Published:** 2025-09-23

**Authors:** Asger Kirkeby-Hinrup, Andreas Stephens, Aron Balogh Sjöstrand, Morten Overgaard

**Affiliations:** ^1^Department of Philosophy, Lund University, Lund, Sweden; ^2^Aarhus Universitet Center for Funktionelt Integrativ Neurovidenskab, Aarhus, Denmark; ^3^Aarhus Universitet Institute for Klinisk Medicin, Aarhus, Denmark

**Keywords:** consciousness, theory comparison, theory convergence, neural correlate of consciousness, NCC, empirical evidence

## Abstract

Which of the many available theories of consciousness should a newcomer to the field choose? We consider possible ways to deal with this conundrum. We argue that convergence of theories is unlikely. Next, we consider ways comparing theories highlighting significant issues with existing endeavors in this regard. Given the nature of the field, presumably empirical support has a critical role to play when assessing theories. We examine a selection of *hot topics*—widely debated cases—and conclude that despite these supposedly exemplifying the best possible conditions for progress, they all struggle to move forward debates between theories. This leaves the large amounts of proposed evidence that never became hot topics, the so-called *cold cases* as a candidate to guide us in the conundrum. However, the lack of insight into the number of these *and* the lack of quality control as to whether each was in fact applicable to any given theory, is akin to a replication crisis. Irrespective of the conundrum, this looms large over any attempt to assess and compare theories according to empirical plausibility. There is a simple remedy for this: reduce the number of cold cases through independent assessment. Finally, we explore if a way out of the conundrum is to reject the need to choose between theories and consider proposals that reject the “theory-based” approach to consciousness studies.

## 1 Introduction

Judging by publications and engagement, the study of consciousness is going well. Interdisciplinary ventures abound. Technology, methods, and paradigms for empirical investigations are continuously developed and refined. A large number of researchers work enthusiastically to develop their preferred theory of consciousness and bolster it with empirical support.

In one sense, largely these endeavors have been successful, and a newcomer to consciousness studies now has the luxury of two dozen (or so) viable (i.e., well developed with at least some proposed empirical support) theories of consciousness to choose from. The question now is: Which one to choose? This is the conundrum: What is the right choice? The contemporary field of consciousness studies is still far from able to answer this question. Indeed, we have yet to get over the hurdle of even agreeing on the parameters for an answer. Disagreements on foundational issues, such as what a theory should explain, prevent this question from even getting off the ground. Furthermore, our troubles with answering this question are exacerbated because there are so many theories on offer. At the same time, the large number of plausible theories available makes answering this question more urgent.

Consequently, in recent years, focus is increasingly shifting toward ways to assess and compare theories (Chis-Ciure et al., 2024; Del Pin et al., 2021; Doerig et al., 2020; Ellia and Chis-Ciure, 2022; Ferrante et al., 2023; Kirkeby-Hinrup, 2024a,b; Kirkeby-Hinrup and Fazekas, 2021; Kozuch, 2024; Melloni et al., 2021; Mudrik et al., 2025; Overgaard and Kirkeby-Hinrup, 2021; Sattin et al., 2021; Schurger and Graziano, 2022; Signorelli et al., 2021; Yaron et al., 2021, 2022). This shift in focus indicates that we, as a field, are becoming aware that our current trajectory—in which the number of theories keeps increasing—poses a challenge.

In sum, at the current state of our field, we have an abundance of theories and no good way to decide between them. Burton Voorhees already observed this more than two decades ago with the words: “In Kuhnian terms, the situation is pre-paradigmatic. There are a number of alternate theories that have been proposed, each offering insights, but also suffering from serious defects. No one of them has achieved acceptance as paradigmatic” (Voorhees, 2000) and the number of theories have continued to increase since then. Scientifically, this is not a very desirable state to be in as a field. Supposedly, if we have scientific aspirations, our goal is for the field to be in the opposite state, i.e., to have very few (or one) theories and good ways to decide between them (i.e., test them).

*Prima facie*, there are four ways out of this conundrum. One possibility is that somehow unequivocal evidence about consciousness and its relation to the brain emerges and settles the debates once and for all. Another possibility is that theories start to converge, for instance in response to incoming (but not unequivocal) evidence. A third possibility is to deploy ways to assess and compare theories. Finally, the fourth possibility is that the field moves away from the theory-based approach to studying consciousness.

Strictly speaking, the first possibility is not as much a way *out* of the conundrum, but rather where we would find ourselves *once* we are out of it. In any case, it is unclear what concrete work could be done (that did not rely on pursuing the three other possibilities) in pursuit of this possibility. Nevertheless, it *is* a possibility to hold out hope for a *eureka moment* solution to our conundrum. In the meantime, the three other possibilities do afford concrete action in pursuit of our goal of understanding consciousness. Consequently, these will be the focus in the rest of this paper.

In the next section, we argue that convergence is unlikely. In Sections 3, 4, and 5 we consider how we evaluate theories and the role of empirical evidence. Following that, in Section 6, we suggest that the lack of attention paid to large swaths of empirical evidence undermines the role it can play regarding assessment and comparison. This is akin to a replication-crisis. The upshot of Sections 2 through 6 is that the theory-based approach to consciousness studies is undermined. This leaves the fourth possible way out of our conundrum: moving away from the theory-based approach (i.e., rejecting the need to choose/the premise of the conundrum). In Section 7, we consider some ways of going about this. Finally, in Section 8, we offer some concluding remarks. For an overview of the paper, see Table 1.

**Table 1:** Schematic overview of the paper's structure.

**Possible solution**	**Conclusion**	**Section**
1) Unequivocal evidence emerges and settles the debates once and for all.	Not so much a way *out* of the conundrum, but rather where we would find ourselves *once* we are out of it.	N/A
2) Theory convergence.	Differences are so prevalent and encompassing that convergence of theories is implausible.	2
3) Empirical evidence and ways to assess and compare theories can eliminate theories.	
• Theory comparison	There are significant issues for each of the surveyed approaches with respect to changing people's minds.	3
• Hot topics: The most prominent cases of empirical evidence	None of the Hot Topics surveyed show much promise.	4
• Cold cases: The huge amount of empirical data that receives little attention	The lack of attention paid to large swaths of empirical evidence undermines the role it can play regarding assessment and comparison. This is akin to a replication-crisis.	5, 6
4) The field moves away from the theory-based approach to studying consciousness.	All the alternatives surveyed may be promising, but most are underdeveloped.	7

Left: Possible solutions to the problem: there are too many theories and no good way to decide between them. Middle: Outline of our conclusions. Right: Relevant sections.

## 2 Convergence is unlikely

A major obstacle in the debates between competing theories of consciousness is that theories operate with fundamentally different conceptions of the explanandum, i.e., there is significant disagreement about how to conceive of consciousness.

To this one might object that there at least seems to be agreement that the phenomenon is best described by reference to the work of Thomas Nagel and David Chalmers. This claim is supported by the fact that in the introduction to almost all publications in consciousness studies, the explanatory target is defined either by reference to there being *something it is like* to be in a conscious state (Nagel, 1974), or cached in relation to “the hard problem” (Chalmers, 1995), or both. Appeals to intuition, or common-sense are also not uncommon, such as defining consciousness by reference to its absence; for example, when (Tononi 2008, p. 216) claims that “Everybody knows what consciousness is: it is what vanishes every night when we fall into dreamless sleep and reappears when we wake up or when we dream.” So, at least, there may seem to be a consensus on a delineation of the phenomenon in the above ways. However—despite surface appearances—there is reason to think that even what consensus there is may be merely linguistic, given that there is widespread disagreement about what exactly *what it is like* amounts to (Block, 2011a,c; Rosenthal, 2011; Weisberg, 2011), “how much” there is of it (Block, 2011b; Knotts et al., 2019; Kouider et al., 2010), what we can take from how it subjectively appears (Kirkeby-Hinrup, 2023; Schwitzgebel, 2008), where to look for it (Boly et al., 2017; Lau and Rosenthal, 2011; O'Regan, 2012; O'Regan et al., 2005; Solms, 2014, 2019, 2021; Solms and Friston, 2018), what it takes to solve the hard problem (Majeed, 2016; Mills, 1996; Robinson, 1996), what theories of consciousness (should) attempt to explain (Doerig et al., 2020, 2021), and what counts as an explanation (Schurger and Graziano, 2022). Put differently: as a field, we *do* agree that there is something about which we can know something (i.e., we agree that there *is* a phenomenon). But we do not agree on the characteristics of the phenomenon or the parameters for investigating it. That is; we do not agree on what the explanatory target is. Consequently, we do not agree on what a theory should explain.^1^

One positive upshot of this has been interesting endeavors to map the various answers to these questions and correlate them with theories (Chis-Ciure et al., 2024; Sattin et al., 2021; Seth and Bayne, 2022; Signorelli et al., 2021; Yaron et al., 2021, 2022). While these endeavors are of great value when trying to understand individual theories, as well as differences between theories, each at the same time exemplifies the radical differences that exist concerning how to conceive of the explanandum. Let us consider briefly two examples. Firstly, in Sattin et al.'s (2021) review of the dimensions along which theories diverge (covering the years 2007–2017), they found at least 29 distinct theories of consciousness. While there were pairwise similarities, each of these theories operated with a unique working definition and/or conception of consciousness. Secondly, an overview of theories done by (Signorelli et al. 2021) organized theories of consciousness into a 3-dimensional model (see fig. 2:7 of Signorelli et al., 2021). The first dimension tracked whether a theory focuses on what makes a state conscious, or the experiential quality of conscious states. The second dimension tracked whether a theory pursued a unificatory or mechanistic explanation. The third dimension tracked whether a theory pursued a functional or causal explanation. The position of a theory along these three dimensions defines a unique point in the 3-D space, and when plotted together the differences between theories become manifest. The points are scattered across the 3-D space, with many located in the opposite end to others, and a disproportionate number of theories being situated toward the extreme end of at least one dimension.

One way to illustrate how the findings in these reviews impact the probability for convergence is by considering whether the various conceptions of the explanandum revealed in these reviews are even translatable into each other. The issue at stake is excellently articulated by Amerio and Colleagues who propose to:

“[…] imagine two researchers, Scarlett and Amber, who study the phenomenon of “pinkness.” Scarlett uses a design space that has three dimensions, corresponding to the three base colors of the RGB system (i.e., red, green, and blue). After experimenting with various color combinations, she identifies the area of RGB space in which the color pink is produced. Amber, however, defined her experiments in the YMCK color space. How can Scarlett and Amber's experiments be integrated in a single design space? Two conditions should be met. First, the definition of what “pink” is must be shared between the two scientists. If the range of colors that Amber classifies as “pink” is wider than Scarlett's, then mapping the results of their experiments onto each other is meaningless.” (Amerio et al., 2024, second paragraph).

In this analogy the conceptual differences found in the above reviews of the explanandum (consciousness) correspond to different definitions of pink or incommensurable scales for measuring pinkness. If the analogy holds the upshot is that translating theories into each other is meaningless, and we should temper expectations of convergence.

In a similar vein, (Evers et al. 2024) considered commensurability between theories of consciousness on four nested levels. While they do find commensurability between subsets of theories (along particular dimensions), there is also significant heterogeneity between theories and across the whole set. For instance, they conclude that:

“[…] although some theories may seem logically commensurable at first glance, sharing some conceptual and even empirical similarities, vast gaps nevertheless exist in other domains, for instance in the further specification of consciousness and the approach to testing and verifiability. In other words, logical commensurability does not imply or guarantee conceptual similarity, and does not exclude vast empirical differences.” (Evers et al., 2024, p. 13).

Furthermore, Evers, Farisco, and Pennartz only consider a relatively small set of theories along a handful of dimensions, but if current data is any indication, heterogeneity in the set of theories would persist (or increase) with the addition of more theories and/or dimensions.

Encouragingly, these significant issues have not dissuaded attention to the *similarities* between theories (Wiese, 2020), or considering the possibilities of convergence (Evers et al., 2024; Storm et al., 2024). To elaborate, Wiese proposed a Minimal Unifying Model (MUM) of consciousness. MUM is unificatory in the sense that it tries to highlight and combine what most theories state as necessary conditions for consciousness. Wiese suggests *information generation* as a possible foundation for MUM given that this concept appears compatible with a broad range of theories. However, the proposal of information as the key unifying feature in MUM also has been criticized (Evers et al., 2024).

In any case, with respect to convergence—and in face of the significant differences between theories discussed above—it is an open question whether the various theories will abandon their unique conceptions of the explanandum (i.e., what they think “consciousness” means; something fairly central to a theory) to jointly pursue a middle-of-the-road alternative with (supposedly) important differences between theories washed out (also thereby possibly reducing explanatory power). Critically, convergence must be a social endeavor, in the sense that more than one theory must be involved. When it comes to convergence, it takes (at least) two to tango, as it were. This points to a further issue, namely that in the context of our conundrum, convergence must also be such that it results in *fewer* theories. It is not enough that two theories converge, work on each theory must also cease, otherwise the theory resulting from convergence is just a new theory, further adding to the plethora of existing theories, taking us further away from solving the conundrum.

In light of the above, it is worth remembering that if *what* theories attempt to explain differs, there is a sense in which they are not theories about the same thing. Yet, the prevalent assumption in the field is that the various theories of consciousness are mutually exclusive. Indeed, this is what fuels the conundrum. In response to this, it might be highlighted that it is not uncommon that theories about distinct things are mutually exclusive. For instance, famously, general relativity is incompatible with quantum mechanics. However, this comparison is a little misguided, because general relativity and quantum mechanics do not purport to be about the same thing, in the way the competing theories of consciousness do. A better analogy would be a case where theories, supposedly about the same phenomenon, disagree about the nature of said phenomenon. One such example is in research on models of the atomic nucleus, where Margeret Morrison says: “[…] we have a case of underdetermination in the extreme. […] this is enhanced in cases where the successes of one model rest on exactly the assumptions that are contradicted by others” (Morrison, 2011, p. 344). This seems very similar to the situation in consciousness studies. Speaking directly about convergence, Morrison notes that “[the models] sheer number and diversity prevents us formulating any clear picture of how the models might converge to a coherent account and we are left with little reason to give credence to any particular model or group of models” (Morrison, 2011, p. 351). The situation described does sound bleak but there is reason to think the situation in consciousness studies is slightly worse yet. With respect to nuclear models, the scientists involved at least agree on *what* to measure in the world, *where* to look for it, and *how* to measure it. They “just” disagree on what the measurements mean, and which is the right model to account for them. In consciousness studies, we have no way of measuring consciousness, i.e., there is no agreement on *what* to measure, or which empirical techniques can be applied to measure it. Possibly, this is partly a result of the disagreements on the explanandum illuminated above.

Moreover, given the significant differences in how consciousness is conceived, there is one further obstacle to convergence between theories. This obstacle is socio-scientific; it requires *someone* to change their mind. Due to an eye-opening quote from Daniel Kahneman (“no one changes their mind,” as communicated on stage by the presenters of the COGITATE results at the much-anticipated event during the ASSC conference in New York in 2023) this issue has attracted attention recently. Subsequently, in writing, the COGITATE group themselves also mention the challenges in changing people's mind (Ferrante et al., 2023). Later, (Kirkeby-Hinrup 2024b) highlighted a concomitant issue, namely that we do not even know what kind of evidence it would take for someone to change their mind, and furthermore that it is likely that this is ultimately arbitrary to the particular researcher. Importantly (and unfortunately) this issue is also applicable to the third (empirical evidence) and fourth (moving away from a theory-based approach) possible ways out of the conundrum. In any case, given the issues discussed in this section, we submit that convergence of theories is nowhere on the horizon in our field.

## 3 Theory comparison

In the previous section, we demonstrated differences in the field regarding how to conceive of the explanandum (consciousness). We suggested the differences are so prevalent and encompassing that convergence of theories is implausible. In this section, we turn our attention to the third possible way out of our conundrum: assessment and comparison of theories.

To establish an initial mutual ground, we limit our scope to positions compatible with consciousness being *naturalized*. On this view: consciousness is supposed to depend on measurable activity (which the field agrees almost certainly involves the brain in some capacity). Roughly speaking, due to the nature of the field, researchers in interdisciplinary consciousness studies will largely agree to this presumption. While this agreement still falls significantly short of agreeing on *what* to measure as well as *how* (like in the nuclear models), it is at least something. Therefore, the idea that empirical evidence can play a special role in moving forward the debates enjoys some consensus between researchers who disagree on almost everything else (cf., the above).

The increased focus on empirical corroboration ever since (Crick and Koch 1998) established consciousness studies as a respectable and empirical science has sometimes been called the *empirical turn*. The underlying assumption is that the explanatory and predictive power (in the empirical domain) of a theory is key to determining its plausibility, and that the most plausible theory is preferable. From this consensus, minor disagreements immediately appear, such as questions about the extent of the brain's involvement and which non-brain factors there may be (Bayne, 2007; O'Regan et al., 2005; Solms, 2019, 2021; Solms and Friston, 2018).

In parallel, the incommensurable definitions of the explanandum discussed in the previous section reappear as an issue for assessment and comparison. In the previous section we cast this incommensurability in terms of an obstacle to convergence, but in the context of assessing and comparing theories it takes the form of a methodological problem. The root of the problem is that across the field there are significant overlaps in the vernacular of supposedly incompatible theories. Put differently, most theories use the same words but have widely different understandings of the concept corresponding to a given word. The most prominent example of this is the word “consciousness” itself. More or less every theory in the field deploys the word “consciousness,” but (cf., the previous section) they do not agree at all about what it means. Similar problems are found regarding many (maybe most) key concepts in the field (e.g., attention, awareness, metacognition, perception, phenomenal consciousness, qualia). This becomes the root of a methodological problem sometimes called *conceptual bleed*. Briefly, in order to apply empirical evidence to the debates, an interpretation is needed to map the empirical concepts (e.g., eye blinks, BOLD signals, button presses, visibility reports etc.) to psychological concepts (e.g., mental state, perception, experience, decision etc.), but as we saw above, one feature of the commensurability issue is that theories disagree on what these psychological concepts mean, and this disagreement bleeds into the interpretations of the evidence (Kirkeby-Hinrup, 2024a; Kirkeby-Hinrup and Fazekas, 2021). In one sense this is perfectly reasonable. Indeed, we should expect interpreters to deploy the theoretical framework and vocabulary they think best captures the empirical data and the concepts they think best describe and categorize the phenomenon under investigation. Unfortunately, in this specific case, the role we are hoping empirical evidence will play for us is exactly determining what vocabulary and which definitions of the central concepts are right.

Despite the above issues, interesting endeavor's to compare theories are in motion. Therefore, the rest of this section briefly considers four different approaches to comparing theories. The objective is to determine if any of them constitutes a promising way out of our conundrum.

### 3.1 Falsification-type approaches

While not strictly Popperian falsificationism (Popper, 1962, 2005), the “Accelerating Research on Consciousness” (ARC) project deploys a methodology guided by the principle of falsification combined with adversarial collaboration (see e.g., Kahneman, 2003). In short, proponents of competing theories agree beforehand on an experimental set-up on which they predict different outcomes. The experiment is then conducted with the expectation that only one theory should be able to be corroborated, while the other(s) is (partly) falsified.

ARC is still in its early stages with results from only one sub-project (COGITATE) being published so far (Melloni et al., 2023). Nevertheless ARC has, due to the buy-in from prominent researchers advancing competing theories, along with its impressive scope and ambition, attracted a lot of attention. However, significant question marks remain pertaining to whether it will be applicable across the field. One reason is that ARC projects treat only a few theories at a time with each project taking years and being very cost intensive. Faced with more than two dozen theories, this becomes unfeasible in practice. Additionally, it is unclear how to weigh multiple ARC results against each other. To boot, it is standard procedure in science to revise one's theory in light of new empirical evidence, so multiyear multimillion dollar ARC projects may not successfully eliminate theories. This was shown in the results of COGITATE that compared predictions from Integrated Information Theory (IIT) and Global Neuronal Workspace Theory (GNWT). No strong conclusion was afforded by the data and both methodology and interpretation of data was questioned by proponents of the participating theories, especially in areas that went against their preferred theory (Ferrante et al., 2023; Melloni et al., 2023). While further issues with ARC have also been highlighted (Kirkeby-Hinrup, 2024b), there is some cautious optimism about the future of adversarial collaborations. Specifically, that tempered with the proper philosophical framework, adversarial collaborations (in theory) are promising catalysts for individual theoretical refinement and development (Corcoran et al., 2023; Negro, 2024). We acknowledge that adversarial collaboration is still in a nascent stage, and its full potential remains to be seen.

### 3.2 Criteria-based approaches

(Doerig et al. 2020) develop an approach (CRIT) centered around a set of criteria for theories. The idea is to evaluate theories against a set of criteria, with the theory that satisfies the most criteria being preferable over the others. An added benefit is that by situating theories in relation to the same set of criteria, a better overview is made possible, and the strengths and weaknesses of each theory are illuminated.

There are questions as to CRITs ability to deliver an evaluation of the set of theories that is sufficiently fine-grained to make any recommendation in our conundrum (Kirkeby-Hinrup, 2024b). Furthermore, the focus on specific criteria can render CRIT blind to the amount of, and quality of, empirical support that different theories might have. To boot, it is unclear how to select criteria in an unbiased way. Finally, given that the set of criteria are relatively small, ties are likely, and it is unclear how to resolve this in a manner that does not subvert the whole approach (Kirkeby-Hinrup, 2024b).

### 3.3 Inference to the best explanation-type approaches

The third approach to comparing theories is based on the method of Inference to the Best Explanation combined with Bayesian updating and accommodated to track the relations between theories and data. In “Quantification to the Best Explanation” (QBE) (Kirkeby-Hinrup 2024b); cf., (Kirkeby-Hinrup and Fazekas 2021) suggests a four-step process to assessing and comparing theories. The four steps are *assimilation, compilation, validation*, and *comparison*. That is: collect evidence for each theory, compile the sets of evidence, validate the claimed empirical support, and compare theories based on the validated sets. The idea behind this methodology is that by quantifying the validated sets comparisons of the respective empirical support for each theory becomes feasible. Furthermore, QBE can offer focused evaluations examining specific domains (e.g., *credence, replicability* or *scope*) of the evidence proposed in favor of a specific theory along with similar filters on the kinds of evidence factored in. A strength of the method is its generalizability and sensitivity, with fine-grained comparisons across multiple theories posing no problem.

Being underdeveloped, QBE nevertheless faces significant challenges. Primarily, the exact mathematics/statistics behind the theory comparison needs further explication. Furthermore, it is unclear how to resolve any disagreements that may emerge about these mathematics/statistics. While there may be some promise in moving disagreements from a domain plagued by conceptual disagreements (philosophy/consciousness studies. See e.g., Section 2 above, and (Kirkeby-Hinrup 2024a) to a domain with a comparatively more determinate and established conceptual framework (mathematics/statistics), it is unclear if this will be sufficient to achieve any kind of consensus with respect to which theory is preferable. This again, partly owes to the socio-scientific factor mentioned in Section 2 above (changing people's mind).

### 3.4 Measure centrality-based approaches

(Chis-Ciure et al. 2024) Measure Centrality Index (MCI) aims to enable fruitful inter-theory classification that systematically can help evaluate and compare theories. The idea is to map the concepts and measures of theories to determine what (kinds of) data would impact a theory and how much a given piece of data would impact it (Chis-Ciure et al., 2024). By using the MCI classification interface it is possible (ideally) to identify specific empirical measures and rank their importance for different theories. The idea is that knowing the (kind of) data that can impact a theory will be relevant to assessing empirical evidence for or against theories. Additionally, knowing how much a specific piece of data would impact a theory allows for better experimental designs in terms of achieving maximal impact on the debates between theories.

Importantly, MCI is only feasible if comparison is possible in the first place, which, arguably given the above, is not always the case. Specifically, the MCI may face questions regarding conceptual bleed, and how to determine non-biased ways to align disparate conceptual frameworks to allow experiments to be devised. Finally, it is worth noting that there is one sense in which the MCI is not as much a way of assessing and comparing theories, as it is a tool to guide such endeavors. So at least one obvious strength of the MCI is as an auxiliary framework to recommend input (in terms of which measurements may be most impactful on debates) to an assessment and comparison process. For instance, the MCI and the QBE approach discussed above may complement each other.

## 4 Theory assessment: hot topics

Occasionally specific cases where novel findings, interpretations, methodology, or conceptual arguments for a time dominate the debates in the field. We will call these cases *hot topics*. Such hot topics are recognizable by a spike in publications (often along with workshops or symposia and similar synergetic academic activity). Hot topics are interesting because they tend to draw in proponents of competing theories and often involve novel arguments or novel empirical data. As a result, the positions (regarding the hot topic) of multiple theories tend to become clearer. To boot, hot topics often inspire novel empirical work or counterarguments. In sum, hot topics have desirable effects, both in terms of improving the clarity of concepts and theoretical frameworks and in terms of empirical work to address differences between theories. For these reasons one might expect that—if anything—hot topics would be where we could see tangible progress in the debates between theories.

So, while the comparison of theories considered in the previous section is insufficient to get us out of the conundrum, perhaps empirical evidence (and here specifically the hot topics, that seem to have the capacity to move debates forward) may nevertheless be enough to get us out.

One example of a hot topic is the perennial debate about higher-order misrepresentation (which suggests that perhaps first-order states are non-necessary for conscious experience); see, e.g., Block, 2011a,c; Gennaro, 2004; Kirkeby-Hinrup, 2014, 2016, 2020, 2022; Lau and Brown, 2019; Lau and Rosenthal, 2011; Rosenthal, 2011, 2012; Weisberg, 2006, 2010, 2011). Other examples include distinctions between types of consciousness (e.g., discussing whether some conscious content is nonconceptual; see, Brinck, 1999; Jacobson and Putnam, 2016), whether perceptual experience is rich or sparse (essentially a debate about “how much” we experience, e.g., Block, 2011b, 2014; Knotts et al., 2019; Kouider et al., 2010), or whether consciousness contains levels or degrees (pertaining to questions such as whether consciousness is graded or dichotomous, as well as “levels” such as various sleep and comotose states; see, e.g., Barra et al., 2020; Bayne et al., 2016; Overgaard and Overgaard, 2010). Regarding contemporary hot topics, it is safe to say AI consciousness is an ongoing case. Next, to elaborate, we will consider four cases in a little more detail.

### 4.1 No report paradigms

One of the most pervasive and long running debates in the field concerns the distinction between Access (A-) consciousness and Phenomenal (P-) consciousness (Block, 1995, 2007). Call this the Access-Phenomenal Distinction (APD). According to APD, A-consciousness is involved in cognition and behavior, whereas P-consciousness is uniquely experiential. The role of A-consciousness with respect to cognition and behavior presents a problem for the measurement of P-consciousness. This problem is exacerbated by the fact that our best access to the (P-) conscious states of a subject is via subjective reports (i.e., asking the subjects what they experience). This is because conceptualization and verbal reporting are hallmark features of A-consciousness. Against this background, the hot topic considered here relates to how one should measure P-consciousness without the measurement being confounded by A-conscious processes related to reporting.

The central aspect here was the so-called *no-report paradigms* (Block, 2019; Duman et al., 2022; Overgaard and Fazekas, 2016; Schlossmacher et al., 2020; Tsuchiya et al., 2015; Whyte et al., 2022). The idea was to measure neural activity in a binocular rivalry (Frässle et al., 2014; Pitts et al., 2010; Sandberg et al., 2014) paradigm that did not require subjects to report visual changes. When viewed independently and when contrasting the no-report measurements with other (report-based) measurements of binocular rivalry the hope was to identify by subtraction the neural signatures related to the reporting and by removing them arrive at the correlates of P-consciousness.

Critical junctures in the debate surrounding this hot topic include (Tsuchiya et al. 2015) pointing out a possible confound related to pre-conscious and post-conscious activity, and (Block 2019, 2020) highlighting our inability to control for task-unrelated thoughts (A-conscious activity). Despite garnering significant attention, this hot topic (no-report paradigms) thus far appears to have failed to move the needle significantly in the debates involving proponents of APD and their critics. Nevertheless, novel no-report paradigms are still being developed, and in so far as one cares about the study of consciousness, one should root for these developments to eventually produce promising results.

### 4.2 Overflow

Block's overflow claim posits that P-consciousness harbors more information than a subject can access (Block, 2007, 2011b). So, subjects can have P-conscious experiences they are not aware of having. Or put more simply: because the phenomenal domain outstrips the cognitive domain, we experience more than we can access.

As a hot topic, has fueled many theoretical considerations about how the evidence could even apply (Cohen and Dennett, 2011; Michel, 2019; Phillips, 2018). On the empirical side, overflow has been fueled by the Sperling paradigm (Block, 1995, 2007, 2011b; Sperling, 1960). In the Sperling paradigm, a matrix of letters is presented briefly, and subjects subsequently are asked to report which letters they saw. Normally, subjects say that it felt like they saw all the letters, but they are able to report only 3–5 specific letters. However, if, *after the visual stimulus of the matrix has disappeared*, subjects are cued to a specific row in the matrix, they retain the ability to report between 3–5 (despite not knowing which row they would be required to report). This indicates that information about the identity of all the letters is available prior to the report, supporting the subjects' claim that they see all the letters. In deploying APD and overflow to explain these results it is posited that subjects are P-conscious of all the letters, but because A-consciousness has limited capacity, not everything in P-consciousness can be accessed. In other words, the 3–5 item caps are not indicative of the amount of P-conscious content.

The hot topic gained new steam with the publication of the color diversity paradigm (Bronfman et al., 2014; Usher et al., 2018), which improved on Sperling by extracting task-irrelevant color-judgments supposedly indicating P-consciousness. In this variant, the letters in the matrix are of different colors, and the subjects are subsequently tasked with judging whether the diversity of colors was “high” or “low.” Despite attention being allocated to the Sperling task, performance was good on the color diversity judgments even for the un-cued rows.

Framing the support overflow derived from the color diversity data in contrast to workspace theories, Block (2014, p. 446) concludes that the color diversity data “reveals that there must have been conscious awareness of specific colors beyond the limits of the global workspace because a trace of that conscious awareness in the form of a diversity judgment can enter the global workspace for free.” Similarly, Jacobson says (2015, pp. 1032–1033):

“[...]the number of experiences of individual colors to which the subject has access is subject to the familiar limitations of working memory – about three or four items; but the relevant judgments of color-diversity can be based on representations—and moreover, on *phenomenal* representations—of many more colors” (Italics from original).

As an excellent example of how hot topics may yield novel empirical developments, (Amir et al. 2023) devised a paradigm to show P-consciousness without A-consciousness. Subjects were queried about the presence of any experience while a persistent pink noise was playing. Only when the noise stopped were the subjects able to report that it had been present, yet supposedly they must have been experiencing the whole time. The reactions in the field to this paradigm have yet to fully manifest themselves, so it is unclear whether it will constitute progress in the end.

Problematically, the Amir et al. paradigm suffers from the same objection as the Sperling and color diversity paradigms. The general reply to this data by critics of APD has been to highlight that there is an equally good interpretation of the data that does not presuppose APD. According to this interpretation task performance in all three cases is driven by unconscious processes, and the sentiment is that it is superfluous to postulate P-consciousness as part of an explanation. In any case, while overflow perennially resurges as a hot topic, there is so far no indication that progress is made to an extent where anyone is changing their mind.

### 4.3 Levels of consciousness

“Levels” was introduced to account for changes in global states of consciousness in the literature on disorders of consciousness (DOCd). As opposed to conscious contents or “local” states, “global states” refer to the overall state of the system. Global states include, for example, sleeping, dreaming, or being awake, and clinical conditions such as coma, sedation, the *minimally conscious state*, and Unresponsive Wakefulness Syndrome (“UWS”; formerly, “vegetative state”) (Barra et al., 2020, fig 2.1). Increasing research on DOCs challenged the long-standing view that consciousness is a binary matter (either you have it or not), suggesting some states are “more” conscious than others. The prospect of overturning an age-old view on consciousness certainly made it a hot topic. The interpretation has since been challenged, however (Bayne et al., 2016; Overgaard and Overgaard, 2010).

Moreover, the very concept of levels has been called into question as not capturing the complexity of the neural states of DOC patients (Bayne et al., 2016). Evidence suggests that levels (e.g., coma, sleep, wakefulness) cannot be tied to a single neural mechanism, and each patient shows unique characteristics (Overgaard and Overgaard, 2010).

At this point, the discussion surrounding levels has become consolidated in the field and now may be more akin to a sub-field than a hot topic. In any case, it seems that levels are largely orthogonal to debates between theories, and there has been no significant shift in opinion with respect to which theory is more plausible in light of discussions of levels. Nevertheless, since levels of consciousness are of particular societal concern (e.g., in terms of recovery predictions for various stages of coma), evaluating and comparing theories in this regard should carry at least some force. Moreover, since most theories agree that dreams are genuine experiences, NREM sleep and dreaming do provide interesting contrast cases, against which to evaluate putative neural correlates of consciousness.

### 4.4 AI consciousness

One contemporary hot topic is AI consciousness. Considerations about (the possibility of) AI consciousness have quickly become a prominent public and scientific concern. The field of consciousness studies, naturally, is engaged with these concerns.

Roughly, there are two overall approaches to assessing AI consciousness. The first approach assumes that some X is necessary for consciousness and then proceeds to check if AI has X. Competing theories of consciousness propose competing candidates for X. Consequently, if we presume any single theory (ideally) this establishes an X and allows us to examine AI systems and draw conclusions about AI consciousness (Butlin et al., 2023). Unfortunately, assuming another theory—thereby establishing a different X—may yield a conflicting conclusion about AI consciousness. To solve this issue, an argument is needed to motivate picking one theory over another, otherwise any conclusions about AI consciousness will be arbitrary (and, for example, unsuitable to guide future research and ethical or political decisions). This means that to solve the issue with conflicting inferences from different Xs it is now necessary to determine which theory of consciousness is “right” so trying to establish this leads us back to the conundrum.

The second approach to assessing AI consciousness is to subject them to so-called *consciousness-tests* or “C-tests” (Bayne et al., 2024). A C-test checks if AI has a certain property or ability associated with consciousness. Typically, a barrage of C-tests is applied to ameliorate a central shortcoming of the approach (Bayne et al., 2024; Butlin et al., 2023). However, with respect to the core idea of the test-approach it is inconsequential if a single C-test is applied, or if a barrage of tests is deployed.

One obvious example of a property or ability associated with consciousness is expressions of consciousness, so-called C-expressions (Kirkeby-Hinrup and Stenseke, 2025). Overall, one would expect C-expressions to be highly correlated with the presence of consciousness, since systems without consciousness would have little reason to produce them, while systems that are conscious would have *some* reason to produce them. This means that if something tells us it is conscious (a paradigm C-expression), then this lends support to the belief that it might be. Yet, a lack of C-expressions does not entail a lack of consciousness. Similarly, the presence of C-expressions does not entail the presence of consciousness. Consequently, to bolster our credence in whichever conclusion we reach, it is useful to add further C-tests. Supposedly, the more C-tests that agree, the higher our credence should be that AI is or is not conscious (Bayne et al., 2024).

This test-approach has similarities with at least two long standing issues in philosophy. Firstly; the issue of induction (Roughly: that sequences of observations never yield certainty. See e.g., Hume, 2000; Minnameier, 2010). Depending on one's leanings with respect to metaphysics and philosophy of science, this may be an acceptable bullet to bite if our credence in the answer (i.e., number of C-tests passed or failed) was very high. In any case, “knowledge” may require something less than certainty (Olsson, 2007, 2016). The second similar issue is the problem of other minds (Kirkeby-Hinrup and Stenseke, 2025), the crux of which is that we cannot *know* if persons (other than ourselves) really are conscious, as opposed to just behaving as if they are. This is essentially the same situation we find ourselves in with the barrage of C-tests. Unfortunately, unlike the problem of other minds, which we can largely ignore by adopting a polite convention that “everyone thinks” (Turing, 1950), we are actually interested in reaching conclusions with respect to AI consciousness. Consequently, to solve this issue we need a C-test that *actually* measures consciousness (a *Real-C-test)*, rather than a barrage of ones that are merely correlated with consciousness (behavior). However, to obtain a Real-C-test requires us to understand how consciousness is generated. The only place to look for this understanding is in humans, since after all, humans are the only beings we are certain are conscious (inferring from our subjective case, c.f. the problem of other minds). This means that a test approach to AI consciousness requires us to understand human consciousness, i.e., have/establish a theory of consciousness. This, again, leads back to the conundrum.

In sum, the two central approaches considered here each faced issues which in turn required a solution to the issues discussed in Section 2 and 3 concerning comparison of theories. In any case, given that progress in AI consciousness seems to rely heavily on progress on human consciousness, there is little indication that this hot topic will move forward debates between theories of consciousness significantly.^2^

### 4.5 Summary

In Section 3, we considered four prominent approaches to comparing theories of consciousness. We were able to identify shortcomings for each of these. The conclusion was for every approach either principled and/or practical obstacles made it unlikely that it would get us out of our conundrum. In this section, we considered cases where interest spiked with respect to certain data, a concept, or a debate, what we called *hot topics*. The motivation for this was that hot topics are characterized by increased levels of engagement, novelty, and progress. Furthermore, because they tend to engage proponents of more than one theory, they offer rare opportunities for truly dynamic exchange and interaction which (all else being equal) is presumably a good thing. Regrettably, in the context of making progress in debates between theories, the example cases considered above hardly moved the needle.^3^ This is unfortunate given that the hot topics were supposedly ideal for making progress. Consequently, with respect to guidance concerning which theory is most plausible, our most prominent approaches to comparison of theories and our best cases of empirical debates to date have yet to yield any progress.

In reaction to this, perhaps too much focus and expectation is put hot topics. Perhaps it is the large amount of steady work outside of the limelight where the progress will be made by researchers quietly grinding away. Because this work is conducted largely outside of the limelight, and much of it receives undue little attention, we will call this domain *cold cases*. In the next section, we will consider this domain and a critical upshot of it.

## 5 Theory assessment: cold cases

As for the hot topics we considered in the previous section, the field has largely moved on, and/or the hot topic has not moved the needle significantly with respect to guiding the choice between theories. Fortunately, in addition to the hot topics, there are many more cases of empirical evidence being deployed in consciousness studies. As an analogy, perhaps the hot topics are merely the tip of the iceberg with respect to empirical support for theories. If this is so, then *not* factoring in the vast amounts of arguments and data that did *not* become hot topics, when deploying empirical support to guide our decision between theories risks yielding the wrong conclusions.

Before we turn to the cold cases, it is useful to start with some context to properly define the role these cases are expected to play. Given the nature of the field, *ceteris paribus* having empirical support for a theory is a good thing. If we think that empirical support is important for the plausibility of a theory, then proponents of theories should strive to maximize their theory's empirical support. The hot topics were inadequate to tip the scales of empirical support, but, because not every discussion or novel paradigm becomes a hot topic, most empirical support may come from work that receives little attention compared to hot topics. This is true both at the level of theories, where only a few theories are *widely* discussed. For instance, in the macro-survey of theories of consciousness in (Sattin et al. 2021, fig. 2) we see that most of the theories examined are associated with one to three papers across the 10 years that are featured, whereas GNW, IIT, and Quantum theories were associated with many more publications, constituting a large majority of all papers examined in the survey. However, it is also true at the level of specific publications, which is our focus here. Specifically, we are interested in cases where empirical evidence has been proposed for or against theories, but which have received little attention. Specifically, we here take “received little attention” to mean that a case has not been independently assessed. What exactly is meant by *independent assessment* and how the lack of it feeds into an overlooked replication crisis we will return to below. However, before that, it is illustrative to start with an example of a cold case.

### 5.1 A cold case example

In (Smith 2019) argued for the distinction between phenomenal and access consciousness based on hydranencephaly. Hydranencephalic patients are born without either cerebral hemisphere, leaving them unable to move (due to lack of motor cortex) and with short life expectancy. According to Smith, hydranencephalics are phenomenally conscious but lack cognitive access. He bases this on their differentiated affective responses in relation to their primary caregiver vs. strangers. Because the patients are missing (parts of) the cortical areas (such as the prefrontal cortex) thought to underlie A-consciousness, Smith concludes they cannot have A-consciousness in the normal sense. Smith suggests that hydranencephalics may have another kind of affective-grounded access (consciousness) which explains their ability to discriminate between the primary caregiver and strangers.

Here, we will not weigh in on the merits of Smith's argument. This is because our interest is in what makes this a cold case. Namely, on the one hand: that Smith applies an empirical phenomenon to the APD distinction (which is a proxy for a theory), and on the other hand, that this application has received little to no attention (e.g., in terms of citation metrics).

Now, if we think empirical plausibility is important, then cases like Smith's should be counted when we assess and compare theories. However, they generally are not (unsurprisingly since this is part of the definition of “cold cases”). What should one make of this? Starting with a practical perspective one issue is that no-one is *in fact* counting these cases. In other words, there is some actual work to be done. Since this is “merely” a practical issue, we will leave it to the side here, and focus instead on another issue, which is that if we want to count Smith's case when we assess and compare theories, then Smith's case itself must be (independently) assessed.

### 5.2 Independent assessment

Our notion of independent assessment has counterparts in the notion of replication in empirical sciences and fact checking in public discourse. We suggest such work has a crucial role to play, with respect to including cold cases when we assess and compare theories. The plausibility of a proposed connection between a piece of empirical evidence and a theory of consciousness (e.g., whether or not we should count the cold case in question when assessing and comparing the theory) may be impacted by several factors. For instance, there may be alternative (better) explanations of the outcome, alternative hypotheses that need to be tested, more than one cause of the phenomenon, issues with the experimental paradigm, unnoticed less salient outcomes, the conceptual mapping has gone awry, reasoning errors, etc. (Fink, 2015, 2016; Kirkeby-Hinrup, 2021; Kirkeby-Hinrup and Fazekas, 2021; Kozuch, 2014; Malach, 2011; Odegaard et al., 2017; Overgaard and Kirkeby-Hinrup, 2021).

In comparison, the hot topics do not suffer from such issues to a similar extent. Presumably, this is a direct effect of the additional attention hot topics enjoy. Occasionally, the actual debate (that turns something into a hot topic), will be about one or more of these factors. For just two examples of discussions of such factors are the many competing interpretations of change blindness (Block, 2011b; Lau and Rosenthal, 2011; Sergent and Dehaene, 2004; Tononi and Koch, 2008), and discussions of the COGITATE results (Ferrante et al., 2023; Melloni et al., 2023).

As with counting the cold cases, we again face a practical issue when it comes to independent assessment. However, this time around, it is a little more serious. The issue here is *who* performs the independent assessment (not just *getting people to do it*, which was the issue with counting cold cases). Call this the vetting problem. Briefly, the vetting problem concerns who is suitable to carry out the independent assessment of cold cases. It turns on the fact that most researchers knowledgeable enough to evaluate proposed empirical support (or objections) may not be suitable for this task, as they have vested—albeit often different—interests in the debates. In other words, there is a risk that when independently assessing cold cases, sympathizers of a theory may be biased, and objectors to a theory may beg the question. Fortunately, we think this problem washes out if one does not consider the assessment process as a finite one-time exchange with a limited number of participants. Instead, we propose the independent assessment be thought of as a dialogue in the community. In one sense, one might say that independent assessment really is an exercise in turning cold cases into hot topics. Formulated differently, one might say that if we want to count cold cases when assessing and comparing theories, they need to stop being cold cases, and the overall project (if we want empirical evidence to matter, when assessing and comparing theories) is to eliminate cold cases.

Concretely, independent assessment is not hard, as it merely involves scrutinizing the empirical data, its interpretation, and the argument in which it figures. The purpose is to identify if the proposed empirical argument (or objection) suffers from any of the shortcomings above. If no serious issues are found, then we can safely count the cold case when assessing and comparing theories. In case some shortcoming is identified, this is not necessarily negative, as it points to avenues of further exploration to clarify if the shortcoming can be remedied, and, if so, taking steps to remedy it. Ultimately, if a shortcoming cannot be remedied, then the cold case can be discarded and must be disregarded when assessing and comparing theories. Next, we consider two examples of independent assessment.

### 5.3 Independent assessment examples

(D'Aloisio-Montilla 2017a,b) argues that aphantasics' performance on change detection paradigms supports overflow. Aphantasics lack the ability to generate voluntary mental imagery. According to D'Aloisio-Montilla, no-overflow accounts must appeal to an internal image to explain the results of retro-cue paradigms (Landman et al., 2003). The performance of aphantasics is on par with normal subjects, but we cannot appeal to internal imagery in their case. This is generalized to suggest it is unlikely that internal imagery is driving the performance in normal subjects. If we cannot appeal to internal imagery, D'Aloisio-Montilla argues, this supports the competing interpretation, viz. overflow. When independently assessing this argument, (Kirkeby-Hinrup and Fazekas 2021) find that the two empirical paradigms D'Aloisio-Montilla combine to make his argument differ in significant ways that subvert his conclusion.

The second example concerns the possibility of higher-order misrepresentation, which even if a perennial hot topic (Block, 2011c; Brinck and Kirkeby-Hinrup, 2017; Brown et al., 2019; Gennaro, 2004, 2013; Kirkeby-Hinrup, 2022; Rosenthal, 2011, 2012; Weisberg, 2010, 2011), contains cold cases. In one early publication, Hakwan Lau and David Rosenthal proposed the cases of *rare Charles Bonnet syndrome* (Lau and Rosenthal, 2011) as evidence of higher-order misrepresentation (higher-order thoughts that do not have a target first-order state). In “normal” Charles Bonnet syndrome, subjects have visual hallucinations. However, in the rare cases, subjects had damage to the primary visual cortex (Ashwin and Tsaloumas, 2007; Duggal and Pierri, 2002), which Lau and Rosenthal hypothesize underpins the generation of first-order states. The upshot is that there (*ex hypothesis*) cannot be any first-order visual states corresponding to the visual hallucinations in the rare cases of Charles Bonnet syndrome. In sum, supposedly, the cases rare Charles bonnet syndrome demonstrates that higher-order representations without target first-order states are sufficient for conscious experience. In his assessment of the underlying argument supporting this conclusion, (Kirkeby-Hinrup 2014) found an equivocation in one of the premises. Furthermore, neither of the possible resolutions to the equivocation allowed the argument to persist. Consequently, the cases of rare Charles Bonnet syndrome cannot be counted when assessing and comparing the empirical support for higher-order theories (the group of theories for which the possibility of higher-order misrepresentation is relevant).

Each of these two examples found underlying issues with proposals of empirical support. Importantly, the identified issues were not identical. With respect to D'Aloisio-Montilla's deployment of aphantasics' performance on retro-cue tasks to argue in favor of overflow, the issue concerned the interpretation of the empirical paradigms. With respect to Lau and Rosenthal's deployment of the cases of rare Charles Bonnet syndrome to argue in favor of higher-order misrepresentation, the issue concerned the way they structured the inference from empirical data to theoretical claims. Together, these two examples go some way to demonstrate the heterogeneity of issues that may obtain in the application of empirical evidence to theoretical argument. Furthermore, these examples demonstrate the importance of assessing cold cases, since each case resulted in the discovery of an issue that meant we should not count the empirical support in question when assessing and comparing theories.

On a final note, one should not be blind to other positive side-effects of allocating attention to cold cases. While this activity is justified alone by its role in validating evidence, there are also positive upshots in cases where issues are found. Firstly, it prompts proponents of the evidence to reconsider evidence and reconstrue their argument, which constitutes progress. Secondly, when considering collections of cases where issues have been identified it may be possible to extract patterns that may inform us about the viability of certain ways of constructing arguments (e.g., arguments of type X often involve equivocations). Finally, merely getting more eyes on a case may yield interesting outcomes. This means that diverting attention from the hot topics may reveal untapped and fruitful novel perspectives or areas of research; things that would have gone unnoticed without more people attending to a given cold case.

## 6 An overlooked replication crisis

A precondition for being able to arbitrate between competing theories of consciousness on the bases of their empirical support is some form of consensus on their respective amounts of support. To elaborate, the reason we want to assess and compare the empirical support of theories in the first place is because convergence was unlikely, and empirical evidence appeared to be the only viable alternative to guide a decision between theories. Consequently, if there is no consensus on the respective support of theories then any attempt to assess and compare according to empirical plausibility will be contentious, and we are back where we started, with no way out of the conundrum (i.e., no guidance in the choice between theories).

In other words, if we want empirical plausibility to matter, we need to clarify what the respective empirical support *actually is* for each theory. As argued above, independent assessment of proposed empirical support (or objection) serves a critical role in this clarification. To reiterate, some “quality control” is needed on what counts as empirical evidence. The role of “quality control” is played by independent assessment. Evidently, from our example cases above where purported empirical support had issues, this matters.

The need for independent assessment holds for our attempt to count cold cases when assessing and comparing theories. However, it also holds for the field in general given that the one thing (more or less) everyone agrees on is naturalization. There is something paradoxical about believing that consciousness depends on measurable physical activity, i.e., empirical phenomena, yet ignoring large swaths of data (the cold cases).^4^

So, the replication crisis we want to highlight is the apparent number of cold cases. Importantly, we are not claiming that the mere existence of cold cases subverts all comparison. In fact, for reasons relating to scientific communication and publication delays it may seem like some cold cases will *necessarily* exist (for a while, until the independent assessment is done) whenever new evidence is published. Be that as it may, we do think the following two considerations apply. Firstly, we should avoid leaving cold cases too long without assessment (what “too long” entails is left open). Secondly, we should avoid that the total number of cold cases (perhaps as a percentage of total cases) becomes too big (what “too big” entails is left open). In a sense, both are practical considerations pertaining to managing the backlog of proposed empirical support, which in turn amounts to avoiding a new replication crisis. However, before we can get to that point, it is necessary to deal with the current replication crisis. To do this it is necessary to get a clear view of the total amount of empirical evidence proposed in favor of each theory, and independently assess this on a case by case basis (roughly steps 2 and 3 in the methodology proposed in Kirkeby-Hinrup and Fazekas, 2021. However, here we are advocating this independently of their proposed IBE account).

In the above, our tacit assumption is that we (as a field) should want empirical evidence (hot topic or not) to matter, and scientifically speaking it is what we should pursue. If we are right about this, then the overlooked replication crisis is an issue (Importantly, this is not to say that our situation is identical to the one in social psychology that gave rise to the “replication crisis” idiom (see e.g., Meyer and Chabris, 2014 for an overview). Nevertheless, similarities exist, and if we want to apply empirical evidence in assessing and comparing theories, we should not be blind to this situation). Fortunately, it is an issue that has a straightforward remedy, namely independent assessment. Our caution is that without pursuing this remedy—considering the little success in comparison and impact of the hot topics—empirical evidence is undermined to guide us with respect to the conundrum.

Since there are systemic issues related to how we apply empirical evidence for or against theories of consciousness in general, and our best case scenarios (the hot topics) have shown little ability to make progress. Until the replication crisis is dealt with, it does not seem the third possibility (assessment and comparison) is a viable way to figure out which theory to choose. Importantly, as we have highlighted, there are concrete ways of dealing with this situation that merely involves re-allocating attention (time). Nevertheless, until this is done, our expectations to the role empirical evidence in respect to our debates should be tempered.

## 7 Alternatives to the theory-based approach

In this section, we turn our attention to the fourth possible way out of the conundrum. Given that the first three possible ways out (1) hope, (2) convergence, and (3) assessment and comparison— did not appear promising, then perhaps the root of the problem is with the theory-based approach itself or one of its assumptions. Put differently, perhaps it is the idea that theories are mutually exclusive, and we need to choose between them that is the root of our problem. Here, we will consider five alternatives to the theory-based approach. The question at the root of our conundrum was which of the available theories a newcomer to consciousness studies should choose. Each of these five alternatives (for different reasons) deny that theories are mutually exclusive and consequently coincide in rejecting the need to choose between theories. If any of these approaches are viable, then there is a way out of the conundrum.

### 7.1 Theories are not mutually exclusive because they reflect distinct mechanisms that jointly create consciousness

The first alternative we consider is Joint Determinant Theory (“JDT”). At the core of this approach is the view that consciousness is fundamentally pluralistic, and theories need not worry about being right because their proposed processes may all play a role in conscious experience (He, 2023). According to JDT, different conscious contents such as emotions, thoughts, volitions, and perceptions do not rely on one underlying consciousness-generating mechanism. Instead, integrated conscious experience supposedly is the result of the interaction of a variety of content-specific NCCs, each having their own underlying neural substrate.

Consequently, on this view the search for a universal NCC that underpins all conscious states is misguided. Instead, JDT proposes that conscious experience can be jointly created by distinct neural mechanisms. Because of this, a pluralistic approach to the study of consciousness is recommended by He. A natural consequence of this is that we should study content-specific NCCs separately, and that they may require separate explanatory models. As there is less controversy concerning the studying of local NCCs, this would allow us to build a “stronger empirical foundation” (He, 2023, p. 9). This same fact would also allow researchers to converge around clear explananda, as is not the case currently.

However, the approach does not offer the immediate gratification of theoretical accounts, being rather a tentative framework for the continued study of consciousness. As (He 2023, p. 10) notes: “An implication of this pluralistic view is that there may not be a ‘Eureka’ moment that explains all of consciousness and the work ahead may take longer than some may expect.” However, He is cautiously optimistic about whether studying phenomena separately will eventually yield the neurobiological principles of consciousness and proposes JDT as a better foundation for future endeavors to discover them.

### 7.2 Theories are not mutually exclusive because they address different levels of explanation/abstraction and/or different aspects of the same phenomenon

(Mitchell 2002, 2003) proposes the Integrative Pluralism (IP) framework that aims to resolve conflicts between theories using multiple levels of analysis. Mitchell questions whether supposedly competing theories really are about the same thing (see Section 2 on Convergence above). Perhaps thinking they are about the same thing is what makes them appear mutually exclusive. As an alternative, IP suggests that theories provide complementary and compatible explanations.

Mitchell does not see non-commensurability as a problem. Even same-level theories need not conflict—even though they can—due to their different ways of abstracting over the phenomenon. Instead, competing theories can be seen to address different aspects or features of the same phenomenon. *Theoretical* pluralism is, according to IP, justified and so two different same-level models can provide two different perspectives on the same thing.

By integrating different models, a better, more unified, understanding of the phenomenon is possible according to IP. Consequently, a plurality of theories can give a better explanation of a phenomenon by being integrated. So, on any level of description, there should be allowed a plurality of theories, but when multiple levels are integrated it will turn out that only one “series” of theories will be compatible and thus most plausibly considered tentatively true.

### 7.3 The theory-level approach is futile, we should focus on their shared lower-level constructs

The construct first-approach, argues that the way the theory-driven approach bridges the gap between theory and empiricism, for example by ways of formulating NCCs, is inherently problematic (Fazekas et al., 2024). The proposed alternative is instead to focus on the theoretical constructs themselves.

Extracting constructs is done by “deconstructing” existing theories of consciousness. This involves stripping away excessive theoretical infusion to identify “clean” theoretical constructs. The next step is to map these constructs—including the characteristics of their corresponding lower-level empirical constructs—onto a so-called “construct space” (see Fazekas et al., 2024, Figures 1 and 3). As for the latter, these features are the properties at the neural level which signals the presence of the higher-level construct. Mapping these onto a shared space reveals their relationships, for instance if constructs inhabit overlapping regions. This may allow for empirical investigation, for example, if they are partially defined in light of the same neural activity patterns.

Moreover, by highlighting similarities between theories, the approach is hopeful that, over time, more sophisticated theoretical frameworks might emerge that build on insights gained from this “bottom-up” investigation of consciousness. To elaborate, the space might reveal *regions* of the construct space that correlate strongly with the presence of consciousness. These regions in turn may be characterized in high-level terms, thereby unlocking a way to guide the study of high-level constructs using the construct space. In any case, building studies on constructs themselves, instead of proceeding from a theory eliminates the risk of disagreements around *what* needs explaining (see Section 2), since the lower-level empirical constructs supposedly are theory neutral. This approach has also been utilized in a recent study to look at a proposed taxonomy of mental states by their contents (Van den Driessche et al., 2025).

### 7.4 Theories are not mutually exclusive because theories may truly reflect different ways consciousness gets generated (in humans), i.e., more than one theory may be right

The Multiple Generator Hypothesis (Kirkeby-Hinrup et al., Forthcoming) proposes a distinction between principles and generators. Principles denote *ways* consciousness *can* be generated. A Generator instantiates a principle in some physical system. Hypotheses about possible generators can be abstracted from extant hypotheses about the neural correlates of consciousness (Block, 2005; Boly et al., 2017; Fazekas et al., 2024; Ferrante et al., 2023; Fink, 2016; Francken et al., 2022; Koch et al., 2016; Lepauvre and Melloni, 2021; Overgaard et al., 2020; Overgaard and Overgaard, 2010). Similarly, hypotheses about principles can be extracted by analyzing extant theories in terms of sufficiency claims. To illustrate, according to higher-order theory (Rosenthal, 2008, 2012) a higher-order thought is necessary and sufficient for conscious experience (sometimes called “subjective appearance”). Abstracted into the MGH framework, this suggests the following principle: some kinds of metacognitive relation are sufficient for consciousness.

From this, the MGH suggests that—*pace* what appears to be the tacit assumption in the field—perhaps neither principles nor generators need be mutually exclusive. In other words, in case the hypothesized above principle is actually true (if certain kinds of metacognitive relations in fact are sufficient for consciousness) this does not entail that other principles may not *also* be true. Similarly, in case we find NCCs (a generator) corresponding to the hypothesis of proponents of Global Workspace theory (Mashour et al., 2020) this does not entail that there are not *also* other NCCs (generators) in the brain that generate conscious states *independently* of the workspace generator. Consequently, the MGH suggests that one or more principles and/or generators exist. Importantly, the MGH differs from the JDT account discussed above regarding the interaction and independence of generators. On the JDT, multiple NCCs (that also may vary depending on content) co-operate to produce a single conscious state, whereas on the MGH generators are independent of each other, and can each produce a conscious state.

Finally, the MGH remains neutral on whether the mapping between principles and generators is one-to-one or one-to-many. To elaborate, it is left open whether a single principle can be instantiated in physically dissimilar generators (i.e., whether the principle is multiple realizable). Perhaps it depends on the principle, and some principles require specific physical features of generators, whereas other principles are more flexible with respect to how they can be instantiated.

### 7.5 Theories are not mutually exclusive—exactly because of their difference in explanandum—they may converge at neural levels and jointly increase understanding

(Storm et al. 2024) point out that much research within consciousness studies attempts to explain different aspects of consciousness. And many theories seemingly converge—on the neural level—motivating attempts to integrate them in the hope that such a pluralistic methodology can further our understanding of consciousness. That is, the theories need not contradict one another, rather they could, in fact, be compatible and even complement each other concerning the fundamental neural mechanisms and processes. Accordingly, on Storm et al.'s view, consciousness is to be seen as a phenomenon that can include many aspects or subtypes—making the many competing theories in the field, diverging in their focus, strength rather than a weakness.

By stepwise investigating how different theories are similar, and how they differ, on multiple levels of organization, Storm et al. offers a proof of concept concerning the possibility to identify and explore a fruitful integration of theories of consciousness. This methodology is claimed to offer convergence between several theories which offers hopes for integrative future work regarding our understanding of consciousness. However, Storm et al. acknowledge that there still are many gaps remaining—theoretical as well as empirical.

## 8 Concluding remarks

The issue at the heart of the above was the conundrum: which theory should a newcomer to consciousness studies choose? We considered four possible ways to deal with this conundrum. Of these four, we immediately dismissed the first (Hope) as practically uninteresting. In Section 2, we argued that the second possible way (theories converging) was unlikely. The third possible way out of the conundrum had two different aspects. The first (comparison of theories) we considered in Section 3, concluding that there were issues with each of the approaches we treated. The second aspect (assessment of theories) we considered in Sections 4, 5, and 6. First (Section 4), we looked at a range of hot topics and concluded that these did not show promise with respect to moving forward debates between theories. Then we considered the phenomenon of cold cases (Section 5) and suggested that the lack of insight into the number of these *and* the lack of quality control as to whether each was in fact applicable to any given theory, constituted a replication crisis (Section 6). The upshot of these sections was that, since convergence is unlikely, our last resort with respect to answering the question posed by the conundrum is under pressure; namely the idea that empirical evidence may move forward debates. Absent independent assessment, a collection of supposedly relevant *but ignored* empirical evidence will haunt any comparison or guidance. In this case, the significance of empirical evidence for or against theories with respect to our conundrum (by proxy of theory assessment) is significantly undermined.

Concerning our framing of this paper in terms of choosing between theories, one response thus far not considered is: choose for what purpose? (Parker 2020) highlights a relevant aspect of this discussion through her *adequacy-for-purpose* view. In short, she points out that the assessment of which model or theory to choose is, arguably, best evaluated in relation to a specific purpose. It is possible that some disagreements between theories may in fact reflect such differences in purpose. This invites reflection on our actual scientific ambitions, as these may be a driving force in pulling people to a certain theory.^5^

In one sense our considerations above are optimistic. While we have highlighted what we think are significant issues with the application of empirical evidence, the remedy for both issues (1) Getting an overview of cold cases and (2) Independent assessment is simple. With respect to both issues, it is a situation that we, as a field, can literally work ourselves out of. While the solution is plain, there may be other obstacles to its actual implementation. For instance, just like its extra-disciplinary counterparts, replication (independent assessment) may not be the most attractive career building activity for individual researchers. In that regard, novel theories or groundbreaking new paradigms are likely a better pursuit. A similar obstacle may be that researchers often (rightfully and reasonably) are excited about their own theory, paradigm, or aspect, which consequently consumes their work, leaving limited time for these broader issues. In other words, there may be socio-scientific factors, as well as motivational issues at the level of the individual researcher which reduce the likelihood of the replication crisis being remedied.

The fourth possibility with respect to our conundrum was to move away from the theory-based approach. In one sense, this amounted to rejecting the need to choose in the first place (i.e., rejecting the premise of the conundrum). For this way out of the conundrum to be feasible, some alternative must be shown to be viable. In Section 7, we considered a selection of alternatives to the theory-based approach. So far, the viability of each of these remains unclear. Firstly, all the alternatives are still underdeveloped and will need further refinement. Secondly, each of the alternatives would likely benefit from being subjected to independent assessment of the kind discussed above. In any case, with respect to the conundrum, it is encouraging that alternatives do exist.

To summarize, we have suggested that the number of cold cases and the concomitant replication crisis looms large over any attempt to assess and compare theories according to empirical plausibility. We have suggested a simple remedy for this; reduce the number of cold cases through independent assessment. This, at least, would provide a solid foundation for any role empirical evidence plays in assessing and comparing theories. Importantly, it is possible that even if the replication crisis is dealt with, that assessment and comparison is still not a feasible way out of our conundrum (e.g., due to the other issues we have highlighted in this context). Nevertheless, given the shared foundation in the naturalization assumption, we should continue to work on the issues related to the application of empirical evidence. In other words, the endeavors with respect to assessment and comparison can not only co-exist but also likely synergize with explorations of alternatives to the theory-based approach. On a final note, while we have highlighted significant issues with the theory-based approach, it is worth stressing that there is nothing inherently problematic about trying to show that a given theory is right. What is fueling the conundrum is the unargued pre-theoretical assumption that theories are mutually exclusive.

## Data Availability

The original contributions presented in the study are included in the article/supplementary material, further inquiries can be directed to the corresponding author.

## Footnotes

^1^It is worth noting that on some accounts (e.g., Churchland, 1981; Irvine, 2012) the phenomenon itself is denied. Similarly, on some accounts (Birch, 2024) consciousness not seen as a single natural kind, which supposedly counts against a “theory-heavy” approach. We will not delve into these specific considerations here but only highlight that their existence further increases the disagreements about the how to conceive of consciousness. Furthermore, the disagreements mentioned here only cover the relatively narrow sub-domain of neuroscience-focused naturalistic (at least semi-reductionist) theories. The disagreements are exacerbated further if one includes other domains such as phenomenological traditions, enactivism, and eastern philosophy (to name a few).

^2^It is worth noting that many of the issues discussed here in relation to AI consciousness also pertain to other non-human entities such as cerebral organoids and animals. We thank and anonymous reviewer for pointing this out. Our discussion here is limited to AI, since the aim in the present context is only to give an example of a hot topic.

^3^Importantly, our contention here is not that hot topics are undesirable or of little worth. To reiterate: each hot topic is a promising debate, and increased levels of engagement, novelty, and progress are desirable. Our point here is that the track record of hot topics with respect to making progress on our conundrum is not promising. There is no conflict between this and an appreciation for unrelated qualities of hot topics.

^4^One might argue that overall we can trust that even if there are cases where evidence does not hold up to scrutiny, the amount of (unchecked) evidence can still be taken as indicative of the amount of support for each theory. The idea is that overall; the percentage of cold cases with issues will be roughly the same across theories. On this line of thought, we would not need independent assessment because (supposedly) cold cases with issues will not change the relative amount of empirical support between theories. One initial response to taking this as a reason to ignore independent assessment is that it is academically lazy. If we have scientific aspirations, we should care about whether what we count as evidence, *in fact is* evidence. The second is that—without checking—there plainly is no way to know if the percentage of cold cases with issues remains roughly identical across theories. What the evidence shows is that there seems to be no shortage of cases where we check and find an issue with the application of empirical evidence. Above we illustrated this with just two examples, but caution that a much larger sample size is necessary to draw conclusions about the percentages of cold cases with issues for any given theory, let alone the set of theories viewed collectively.

^5^We thank an anonymous reviewer for pointing this out.
